# IMPROVING HOME-CARE SERVICES FOR HIGH-RISK OLDER ADULTS USING PEER-LED VIDEO VISITS TO HOME

**DOI:** 10.1093/geroni/igac059.2229

**Published:** 2022-12-20

**Authors:** Shiv Lamba, Sandra Garcia, Elizabeth Bast, Lauren Penney, Ana Palacio, Sonia Madera, Orna Intrator, Stuti Dang

**Affiliations:** Washington University, St. Louis, Missouri, United States; University of Miami, Miami, Florida, United States; Miami VA Healthcare System, Miami, Florida, United States; South Texas Veterans Health Care System, San Antonio, Texas, United States; Miami VA Healthcare System, Miami, Florida, United States; Miami VA Healthcare System, Miami, Florida, United States; Canandaigua VA Medical Center, Canandaigua, New York, United States; Miami Veterans Affairs Healthcare System, Miami, Florida, United States

## Abstract

Older Veterans at high-risk for institutionalization often require home- and community-based services (HCBS). Yet, current HCBS delivery often fails to meet the needs of high-risk Veterans due to decreased veteran engagement in outpatient programs and limited HCBS capacity. A promising approach to address these gaps is the use of Veteran-Peers to make home-visits. Peer-2-PACT is a peer-led needs-assessment intervention for high-risk older veterans. Two trained peers conducted a checklist-guided virtual and/or in-person home-assessment to identify unmet needs and home-safety concerns. Veterans with access, acceptance and ability for video-capable technology were offered video-visits. We report on the feasibility of video home-visits in this high-risk group, and the experience of the video-visits using the visit-data and interviews with peers.Eight of 27 Peer-2-PACT Veterans successfully completed initial video-visit to home. The video-visit participants (n=8) were age 74±9; Non-Hispanic Black (50%); males (100%), compared to initial in-person home-visit participants (n=19), age 75.3±10.8; Non-Hispanic Black (47%); males (89%). The commonest needs identified during video-home-visits were home-safety devices 5(62.5%), housing assistance 4(50%), and medication refills 2(25%). Peers report that identifying veterans suitable for video-visits was challenging. During video-visits, depth-perception by peers is limited and sometimes needed in-person follow-up. Main advantages of video-visits was ability to identify unmet needs, engage veterans, provide care during COVID, and tele-present to remote clinicians. Preliminary data suggest that peer-conducted video home-visits is a feasible way to identify unmet needs in some high-risk older adults. This is particularly important improve care of Veterans who live at a distance from the facility.

